# Effects of load carriage methods on fall risk and gait variability during stair ascent: a functional data analysis approach

**DOI:** 10.3389/fbioe.2026.1740819

**Published:** 2026-03-11

**Authors:** Xingchen Zhang, Yao Li, Yuling Fang, Hanbing Wu, Danyang Kou, Jingwen Gao, Jiujiang Liu, Yang Sun, Yi Sun, Yuan Gao, Lian Duan, Liang Yu

**Affiliations:** 1 School of Physical Education, Yanshan University, Qinhuangdao, Hebei, China; 2 School of Humanities and Law (School of Public Administration), Yanshan University, Qinhuangdao, Hebei, China; 3 Beijing Sport University, Beijing, China

**Keywords:** functional data analysis, gait, load carrying method, stability, stair walking

## Abstract

**Objective:**

Based on the existing research that predominantly focuses on loaded level walking or employs discrete-point methods to analyze stair negotiation, this study utilizes functional data analysis to systematically investigate the effects of three load carriage methods on gait variability during upstairs walking in healthy adult males, aiming to elucidate the specific neuromuscular adaptation strategies induced by different loading conditions.

**Methods:**

Nineteen healthy young male participants were recruited for this study. Kinematic and kinetic data were collected during stair walking under three load carriage conditions using a three-dimensional motion capture system and force plates. Gait parameters, center of pressure (COP) trajectories, and lower-limb joint angle time series in the sagittal and frontal planes for the hip, knee, and ankle joints were extracted. Functional principal component analysis (fPCA) was employed to reduce the dimensionality and process the joint angle curves, aiming to identify the dominant modes of variability throughout the entire gait cycle. One-way analysis of variance (ANOVA) was subsequently applied to compare between-group differences in gait parameters and COP measures.

**Results:**

Significant differences were observed across different load carriage conditions in step length, single support time, and second double support time (*P* < 0.05). The center of pressure (COP) trajectory in the mediolateral direction also showed significant differences (*P* < 0.05). Regarding joint kinematics, functional principal component analysis revealed significant between-condition differences in the sagittal plane hip angle for principal component 1 (PC1), as well as in PC1 and principal component 3 (PC3) for the frontal plane hip angle (*P* < 0.05). For the knee joint, a significant difference was found in PC1 of the frontal plane angle time series (*P* < 0.05). At the ankle joint, significant differences were identified in PC3 of the sagittal plane angle and in PC1 of the frontal plane angle (*P* < 0.05).

**Conclusion:**

By employing a functional data analysis framework, this study provides a more nuanced understanding of phase-specific compensatory mechanisms during loaded stair ascent, revealing that the shoulder load poses a greater fall risk than hand load. This elevated risk is primarily due to an elevated and asymmetric center of mass, which induces a forward trunk inclination and compromises stability in both the frontal and sagittal planes, necessitating more extensive gait adaptations.

## 1 Introduction

Human locomotion under load conditions embodies a complex process of sensorimotor adaptation, which requires the neuromusculoskeletal system to maintain dynamic stability during the execution of functional tasks (Wang et al., 2013; Boffey et al., 2019). Existing research has systematically elucidated the biomechanical characteristics and alterations in gait parameters during level walking under load carriage conditions (Wang et al., 2023). For instance, hand loads often induce asymmetric trunk postures and modify spatiotemporal parameters, whereas shoulder loads increase spinal compressive forces and alter lower-limb kinematics (Ozgul et al., 2012). Compared to level walking, stair negotiation imposes significantly greater biomechanical demands due to the need for vertical propulsion against gravity. This results in substantially higher lower-limb joint moments, greater energy expenditure, and more challenging balance control—factors that are further amplified under loaded conditions. However, the specific adaptations in walking strategies elicited by different loading modalities during stair negotiation remain inadequately systematized (Conway et al., 2017; Kováčiková et al., 2021; Halsey et al., 2012). Furthermore, previous studies in this context have predominantly relied on traditional discrete-point analysis methods, which extract isolated metrics such as peak values and time-to-peak from kinematic parameters, thereby failing to capture the continuous dynamics inherent to cyclical movement (Richter et al., 2014). Stair walking constitutes a continuous motor task wherein the inherent temporal dependencies across gait cycles are often obscured by cross-sectional statistical approaches. This methodological limitation becomes particularly pronounced when analyzing such activities, thereby hindering a comprehensive understanding of holistic movement patterns. In these complex locomotor activities, subtle yet functionally significant neuromuscular adaptations are likely to manifest throughout the entire gait cycle rather than at isolated time points. Functional Data Analysis (FDA) offers a viable alternative pathway in this context (Lin et al., 2012). This approach treats biomechanical data as continuous functions rather than discrete observations, thereby preserving the inherent temporal structure of movement patterns. It enables the identification of phase-dependent differences throughout the entire gait cycle and quantifies variations in movement quality that may be overlooked by conventional techniques (Su et al., 2017). Currently, FDA has been extensively applied to research in motor development, motor skill analysis, sports injury diagnosis, and the assessment of rehabilitation outcomes (Zhang et al., 2019; Leroy et al., 2018; Warmenhoven, 2024). The core principle of FDA is to treat observed data as holistic functional entities, rather than merely as sequential arrangements of discrete observations (Zhang et al., 2019). In contrast to traditional cross-sectional statistics, this approach not only aligns with the inherently continuous nature of biological systems but also adheres to the principle of finite energy. Although FDA has demonstrated its value across various biomechanical contexts, its application in the study of loaded stair walking remains scarce.

Therefore, this study employs a functional analytical approach to systematically investigate the alterations in lower-limb joint kinematics during upstairs walking under three load carriage conditions (unloaded, hand load, and shoulder load). By integrating gait parameters and stability measures, it aims to establish a refined biomechanical theoretical framework for understanding and assessing dynamic stability and the biomechanical factors influencing fall risk during loaded stair ascent. The findings are expected to provide valuable insights for the design and control of bionic robots. Furthermore, this research may offer preliminary insights that could inform load carriage strategies in daily activities, mountaineering and outdoor expeditions, as well as military operations for populations similar to the one studied.

## 2 Methods

### 2.1 Participants

*A priori* power analysis was performed using G*Power software to determine the minimum sample size required for this study, which was calculated to be 19 participants. Accordingly, a total of 19 healthy young male adults were recruited (mean ± SD: age 18.68 ± 0.86 years, height 182.02 ± 4.35 cm, body mass 70.69 ± 7.85 kg). The inclusion criteria were as follows: 1) good physical health with normal motor function; 2) no engagement in strenuous physical activity or experience of muscle fatigue within 24 h preceding the tests; and 3) no history of significant lower-limb injury within the past 6 months. This study was conducted in accordance with the principles of the Declaration of Helsinki and was approved by the Ethics Committee of Qinhuangdao First Hospital. All participants provided written informed consent after being fully informed of the experimental procedures and potential risks.

### 2.2 Testing protocol

#### 2.2.1 Testing procedure

**FIGURE 1:**
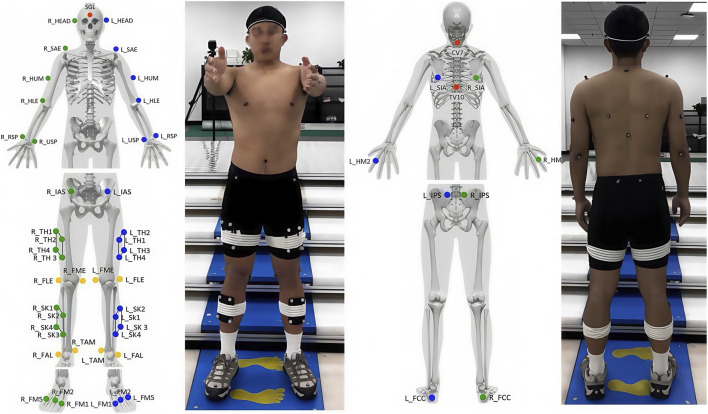
Marker placement.

Upon arrival at the laboratory, participants completed the registration process and provided written informed consent. They subsequently changed into standardized testing attire. The three-dimensional capture volume was calibrated, after which an experimenter affixed reflective markers to predetermined anatomical landmarks (Figure 1). Data collection was then initiated. Kinematic and kinetic data from the lower limbs were synchronously captured using an integrated system comprising four Kistler force plates (sampling frequency: 2000 Hz) and eight Qualisys infrared high-speed cameras (sampling frequency: 200 Hz), connected via an analog-to-digital converter. The hand and shoulder load conditions were chosen to compare two distinct unilateral carriage modes: a low-center distal hand load versus a high-center proximal shoulder load, enabling investigation of how load height and attachment point affect stability during stair negotiation.

**FIGURE 2:**
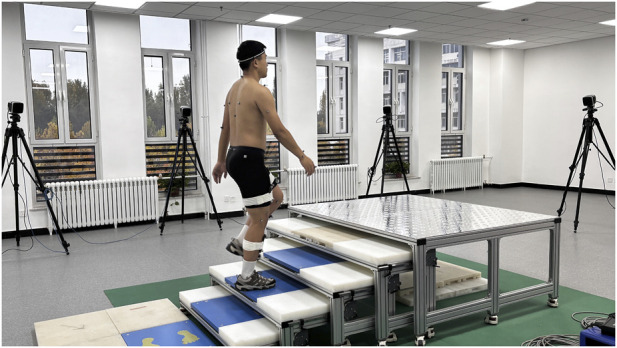
Layout of the experimental site.

The custom-built staircase platform, designed in compliance with the Chinese Code for Fire Protection Design of Buildings (GB 50352-2005), consisted of five steps. Each step had a rise of 15 cm and a tread depth of 30 cm. Four force plates were embedded flush with the walking surface: one was positioned on the level floor preceding the first step, and the remaining three were installed within the recesses of the first, second, and third steps, respectively (Figure 2). The testing protocol required participants to ascend and descend the staircase at a self-selected pace under two load carriage conditions: hand load and shoulder-load, with a total weight of 15 kg. This load corresponds to approximately 20% of the participants’ mean body weight (71 kg), aligning with the body weight percentage standards commonly used in load carriage research. Furthermore, a 15 kg load falls within the typical range (10–20 kg) encountered in daily activities, justifying its selection for this study (Simpson et al., 2012). For each loading condition, ten complete and valid gait cycles were collected. A trial was considered valid only if it was executed without hesitation and with all markers remaining intact throughout the entire motion.

#### 2.2.2 Dominant limb assessment and Gait Cycle Division

**FIGURE 3:**
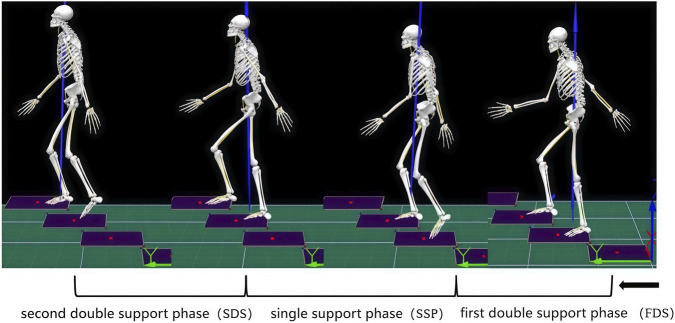
Gait cycle division.

The gait cycle analysis in this study focused on the stance phase of the dominant limb. The dominant limb was determined using a standardized kicking test: a soft soccer ball was placed approximately 2 m in front of the participant, who was instructed to walk forward naturally and kick the ball. This procedure was repeated three times. If the same foot was used to kick the ball in all three trials, that side was designated as the dominant limb. If not, the test was repeated. Throughout the process, no instructions or cues were given regarding the choice of kicking foot. The external load was consistently carried on the same side as the participant’s dominant leg (i.e., an ipsilateral loading configuration). This design was implemented to standardize the biomechanical challenge across all participants, specifically examining the interaction between a loaded upper limb and the ipsilateral (dominant) lower limb during stair ascent. This approach controlled for potential confounding effects of limb dominance asymmetry and allowed us to isolate the effects of load carriage method (hand vs. shoulder) under a common and ecologically valid scenario. The stance phase was further subdivided into the first double support phase (FDS), the single support phase (SSP), and the second double support phase (SDS). The FDS was defined as the period from the initial contact of the right foot on a step to the subsequent toe-off of the left foot from the previous step. The SSP began at the left foot’s toe-off and ended at the initial contact of the left foot on the next higher step. The SDS spanned from the left foot’s initial contact on the higher step to the toe-off of the right foot from the lower step (Figure 3).

#### 2.2.3 Data processing

Gait Data: The Qualisys 2023 software was used to identify the heel-strike and toe-off events during stair walking based on the trajectories of the foot markers. A complete gait cycle was defined as the interval from the heel-strike of one foot to the subsequent heel-strike of the same foot. The software automatically calculated spatiotemporal parameters, including step length, step width, cadence, and walking velocity, from the marker data. The mean coefficient of variation (CV) across gait cycles was calculated using the following formula:

$$\text{STV}=T_{std}/T_{mean}$$ (1)

In Equation 1, $T_{std}$ is the standard deviation of multiple gait cycles from a single participant, and $T_{mean}$ is the corresponding mean value.

The methods for calculating the displacement of the Center of Pressure (COP) are shown in Equations 2, 3.

$${{COP}_{ml}=YCOP}_{\max}-{YCOP}_{\min}$$ (2)

$${{COP}_{ap}=XCOP}_{\max}-{XCOP}_{\min}$$ (3)

In Equation 2, ${COP}_{ml}$ denotes the total COP excursion in the medial-lateral direction, and ${COP}_{ap}$ represents the total COP excursion in the anteroposterior direction.

Kinematic Data: Kinematic data for the hip, knee, and ankle joints in both the sagittal and frontal planes were exported using the Visual 3D software. The kinematic data for each phase were normalized to 101 data points using a cubic B-spline function, resulting in a total of 303 data points across the three phases. This approach was employed to minimize data loss and mitigate the potential obscuring of inherent inter-group differences during the smoothing process (Warmenhoven et al., 2018). Subsequently, functional data analysis was performed using the FPCA toolkit within MATLAB 2022a to process the data programmatically (Ramsay, 2018). Given the cyclical nature of stair walking, the 303 lower-limb joint angle time-series curves were fitted into continuous functions using Fourier basis functions of order 3. In line with previous research (Donoghue et al., 2008), the smoothing parameter was set to 10^^^-7. The resulting functional data were then decomposed via dimensionality reduction into a set of principal components. The mathematical representation for this decomposition is given as follows:

$$\int \upsilon \left(s,t\right)\xi \left(t\right)dt=\mu \xi \left(s\right)$$ (4)

$$\omega \left(t\right)=\xi \left(t\right)\times \sqrt{\mu }$$ (5)

$$c_{i}=\int \xi \left(t\right)x_{i}\left(t\right)dt$$ (6)

In Equation 4, $\upsilon \left(s,t\right)$ is the covariance function of the functional variable, $\xi \left(t\right)$ denotes the eigenfunction, and μ represents the variance matrix of the principal components. In Equation 5, $\omega \left(t\right)$ refers to the weighted coefficient function of the principal components, defined as the eigenfunction weighted by the corresponding eigenvalue. In Equation 6, $c_{i}$ indicates the score of the original functional variable $x_{i}\left(t\right)$ on each principal component. The eigenvalues and eigenfunctions corresponding to the principal components were obtained by solving the characteristic equation of the aforementioned matrix. The number of retained principal components was determined based on the criteria of cumulative variance explained (CVE) reaching 95%, combined with each component’s eigenvalue exceeding 1 (Ryan et al., 2006). To facilitate the interpretation of the principal components, phase variability was eliminated using the varimax rotation method (Jolliffe, 2002) combined with continuous registration (Ramsay and Li, 1998). For the calculation of gait variability (Coefficient of Variation, CV), all ten valid gait cycles per participant and condition were used to compute the mean and standard deviation (Equation 1). For all other analyses—including the extraction of representative joint angle time-series for Functional PCA, and the between-condition comparisons of principal component scores, spatiotemporal parameters, and COP measures—a single gait cycle was randomly selected from the ten cycles for each participant and condition.

### 2.3 Statistical analysis

Data computation and statistical analysis were performed using Excel and SPSS software (version 26.0), while data visualization was conducted with Origin 2021. The datasets were first examined for normality and outliers. Differences in principal component scores, gait parameters, and COP displacement distances across the different load carriage conditions were then assessed using one-way analysis of variance (ANOVA). Post-hoc comparisons, adjusted with the Bonferroni correction, were applied when significant main effects were found. Data are presented as mean ± standard deviation (M ± SD), with a significance level set at *P* < 0.05.

## 3 Results

### 3.1 Differences in gait parameters across load carriage conditions

Results from the one-way ANOVA (Table 1) indicated significant main effects of load carriage condition on step length, single support time, and second double support time (*F* = 6.149, *P* = 0.004, *η* ^*2*^ = 0.185; *F* = 3.948, *P* < 0.001, *η* ^*2*^ = 0.141; *F* = 3.379, *P* < 0.001, *η* ^*2*^ = 0.095 respectively). No significant differences were found for step width, cadence, walking velocity, first double support time, gait cycle duration, or the mean coefficient of variation of the gait cycle (*P* > 0.05). Post-hoc comparisons with Bonferroni correction revealed a significant difference in step length between the hand load and shoulder-load conditions (*P* = 0.004, *d* = −0.756). Furthermore, significant differences in single support time and second double support time were identified between the unloaded condition and both loaded conditions (hand load and shoulder-load) (Single Support: Unloaded vs. Hand load, *P* < 0.001, *d* = −0.079; Unloaded vs. Shoulder-load, *P* = 0.048, *d* = 0.61. Second Double Support: Unloaded vs. Hand load, *P* < 0.001, *d* = 0.077; Unloaded vs. Shoulder-load, *P* = 0.039, *d* = −0.643).

**TABLE 1:** Comparison of gait parameters under different loading modalities.

Gait parameters	No load	Hand load	Shoulder load	*F*	*P*	*η* ^*2*^
Step width (m)	0.120 ± 0.021	0.109 ± 0.027	0.113 ± 0.023	1.192	0.312	0.042
Step length (m)	0.316 ± 0.037	0.327 ± 0.013^C^	0.358 ± 0.057^B^	6.149	0.004	0.185
Cadence (steps/min)	109.077 ± 6.537	111.023 ± 9.360	110.355 ± 10.481	0.238	0.789	0.009
Walking velocity (m/s)	0.647 ± 0.051	0.661 ± 0.070	0.626 ± 0.065	1.518	0.229	0.053
First double support (s)	0.154 ± 0.019	0.151 ± 0.024	0.167 ± 0.022	2.985	0.059	0.100
Single support (s)	0.401 ± 0.036^BC^	0.404 ± 0.039^AC^	0.376 ± 0.046^AB^	3.948	0.000	0.141
Second double support (s)	0.176 ± 0.027^BC^	0.174 ± 0.025^AC^	0.194 ± 0.029^AB^	3.379	0.000	0.095
Gait cycle duration (s)	1.148 ± 0.080	1.119 ± 0.074	1.146 ± 0.086	1.988	0.147	0.069
STV	0.059 ± 0.011	0.070 ± 0.029	0.068 ± 0.049	0.191	0.828	0.025

A: significant vs. unloaded; B: significant vs. hand load; C: significant vs. shoulder-load.

### 3.2 Differences in COP parameters under different load carriage conditions

**FIGURE 4:**
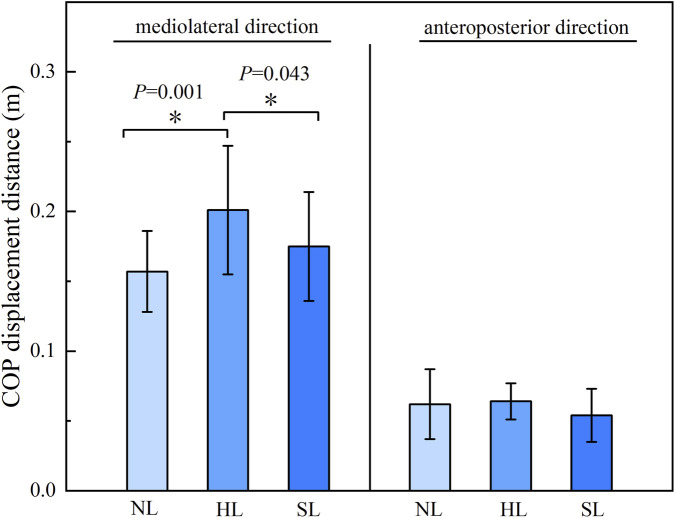
Post-hoc comparisons of COP displacement across load carriage conditions. Note: Left panel shows the total COP excursion in the mediolateral (ML) direction; right panel shows the total COP excursion in the anteroposterior (AP) direction. Conditions: NL, No Load; HL, Hand Load; SL, Shoulder Load. Asterisk (*) indicates a significant difference between conditions at *P* < 0.05.

Results from the one-way ANOVA (Table 2) revealed a significant main effect of load carriage condition on the mediolateral (ML) center of pressure (COP) displacement (*F* = 6.149, *P* = 0.004, *η* ^*2*^ = 0.184). No significant effect was found for the anteroposterior (AP) COP displacement (*P* > 0.05). Post-hoc comparisons with Bonferroni correction (Figure 4) indicated a significant difference in ML COP between the unloaded and hand load conditions (*P* = 0.001, *d* = −1.158), as well as a significant difference between the hand load and shoulder-load conditions (*P* = 0.043, *d* = 0.605).

**TABLE 2:** COP parameters under different load carriage conditions.

Parameter	No load	Hand load	Shoulder load	*F*	*P*	*η* ^2^
ML COP	0.157 ± 0.029	0.201 ± 0.046	0.175 ± 0.039	6.098	0.004	0.184
AP COP	0.062 ± 0.025	0.064 ± 0.013	0.054 ± 0.019	1.437	0.247	0.051

### 3.3 Functional data analysis of hip joint sagittal plane angle time series curves

**FIGURE 5:**
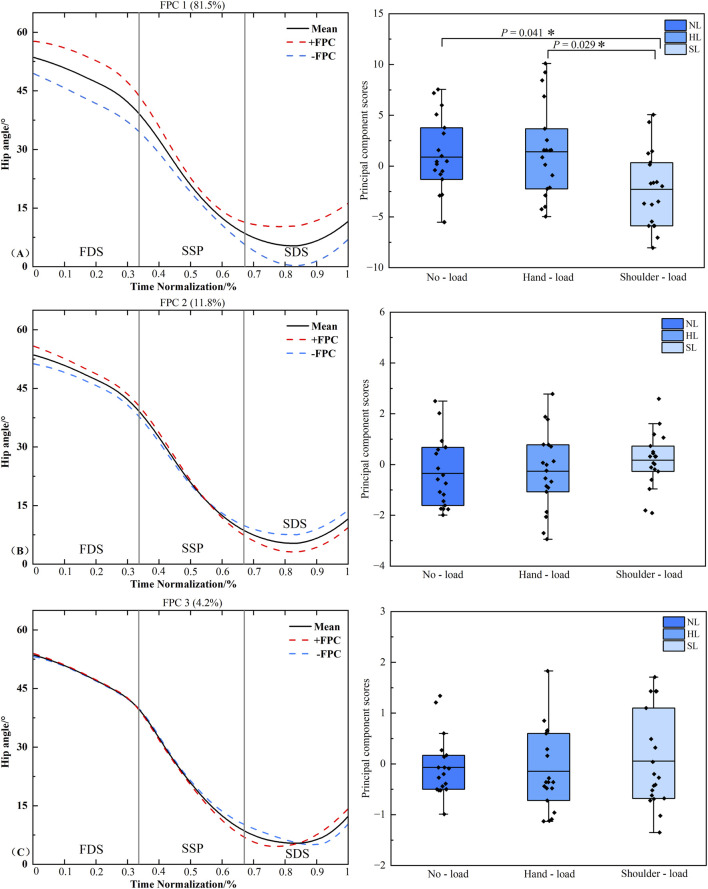
Principal component curves and scores of the hip joint sagittal plane angle. Note: The solid black line represents the mean joint angle curve. The red dashed line (+) and blue dashed line (−) correspond to the mean curve plus and minus an appropriate multiple of the principal component, respectively, illustrating the mode of variability captured by that component. The corresponding box plots on the right display the scores of the principal component for each loading condition, with individual data points overlaid representing each participant’s score. **(A–C)** denote Principal Component 1 (PC1), PC2, and PC3, respectively. Asterisk (*) indicates a significant difference at *P* < 0.05.

Figure 5 displays the results of the principal component analysis for the hip joint sagittal plane angle time series curves under different load carriage conditions. The joint angle time series were reduced to three principal components (PCs) with eigenvalues of 18.265, 2.633, and 0.939, explaining 81.5%, 11.8%, and 4.2% of the variance, respectively, with a cumulative variance explained of 97.5%. The amplitude variation of Principal Component 1 (PC1) for the hip joint angle was primarily concentrated during the FDS and SDS phases. The results revealed a significant difference in PC1 scores between the unloaded and shoulder-load conditions (*P* = 0.041, *d* = 0.839). A significant difference was also found in PC1 scores between the hand load and shoulder-load conditions (*P* = 0.029, *d* = 0.887). No significant differences were detected in the scores of Principal Component 2 (PC2) or Principal Component 3 (PC3) (*P* > 0.05).

### 3.4 Functional data analysis of knee joint sagittal plane angle time series curves

**FIGURE 6:**
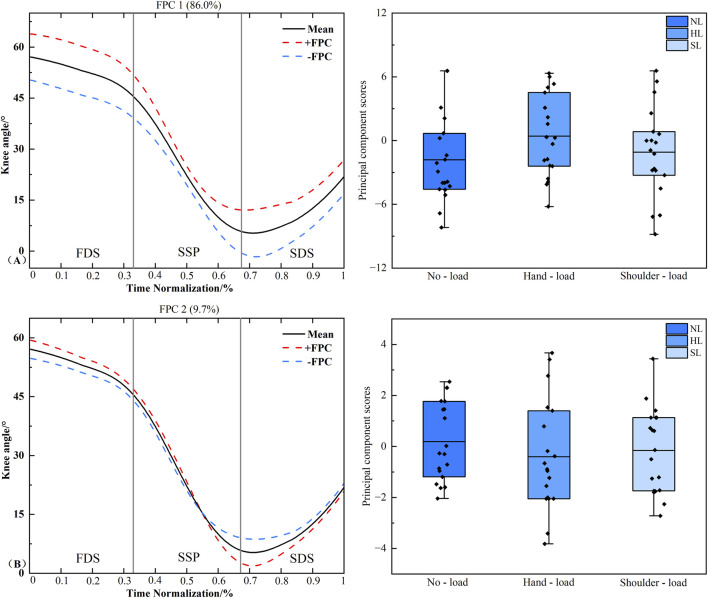
Principal component curves and scores for the knee joint sagittal plane angle. Note: The solid black line represents the mean joint angle curve. The red dashed line (+) and blue dashed line (−) correspond to the mean curve plus and minus an appropriate multiple of the principal component, respectively, illustrating the mode of variability captured by that component. The corresponding box plots on the right display the scores of the principal component for each loading condition, with individual data points overlaid representing each participant’s score. **(A,B)** denote Principal Component 1 (PC1) and PC2, respectively. Asterisk (*) indicates a significant difference at *P* < 0.05.

Figure 6 presents the principal component analysis results of the knee joint sagittal plane angle time series under different loading conditions. The kinematic curves were reduced to three principal components with eigenvalues of 34.946 and 3.924, explaining 86.0% and 9.7% of the variance, respectively, and achieving a cumulative contribution rate of 95.7%. The results indicated no statistically significant differences in the scores of either principal component 1 or PC2 across the different load carriage conditions (*P* > 0.05).

### 3.5 Functional data analysis of ankel joint sagittal plane angle time series curves

**FIGURE 7:**
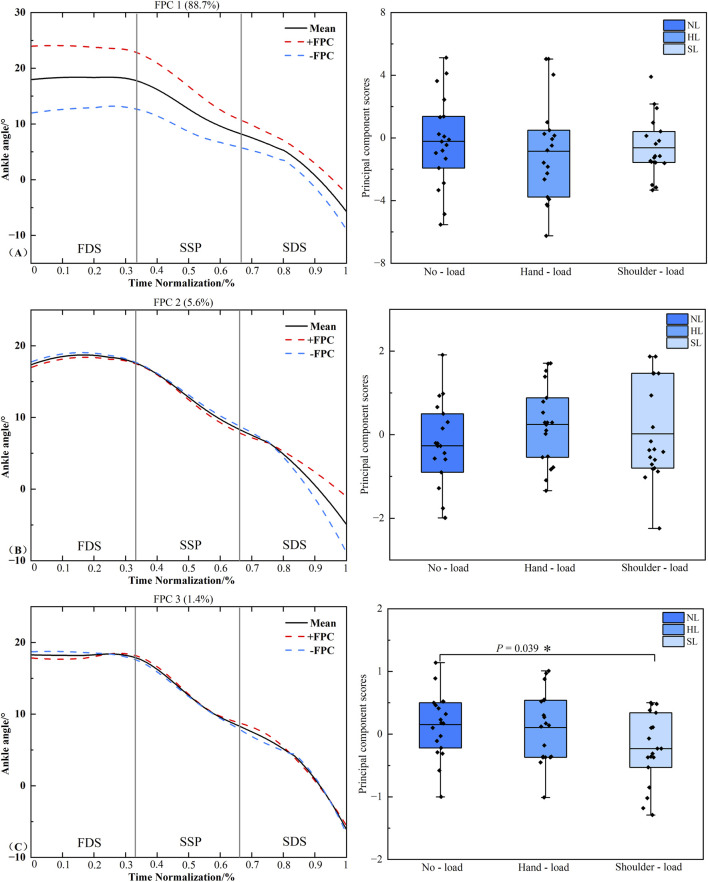
Principal component curves and scores for the ankle joint sagittal plane. Note: The solid black line represents the mean joint angle curve. The red dashed line (+) and blue dashed line (−) correspond to the mean curve plus and minus an appropriate multiple of the principal component, respectively, illustrating the mode of variability captured by that component. The corresponding box plots on the right display the scores of the principal component for each loading condition, with individual data points overlaid representing each participant’s score. **(A–C)** denote Principal Component 1 (PC1), PC2, and PC3, respectively. Asterisk (*) indicates a significant difference at *P* < 0.05.

Figure 7 presents the principal component analysis results of the ankle joint sagittal plane angle time series under different loading conditions. The kinematic time series were reduced to three principal components with eigenvalues of 13.48, 0.944, and 0.298, explaining 88.7%, 5.6%, and 1.4% of the variance, respectively, resulting in a cumulative variance explained of 95.7%. The amplitude variation of PC3 was primarily concentrated during the FDS and SDS phases. Statistical results revealed a significant difference in PC3 scores between the unloaded and shoulder-load conditions (*P* = 0.041, *d* = 0.721).

### 3.6 Functional data analysis of hip joint frontal plane angle time series

**FIGURE 8:**
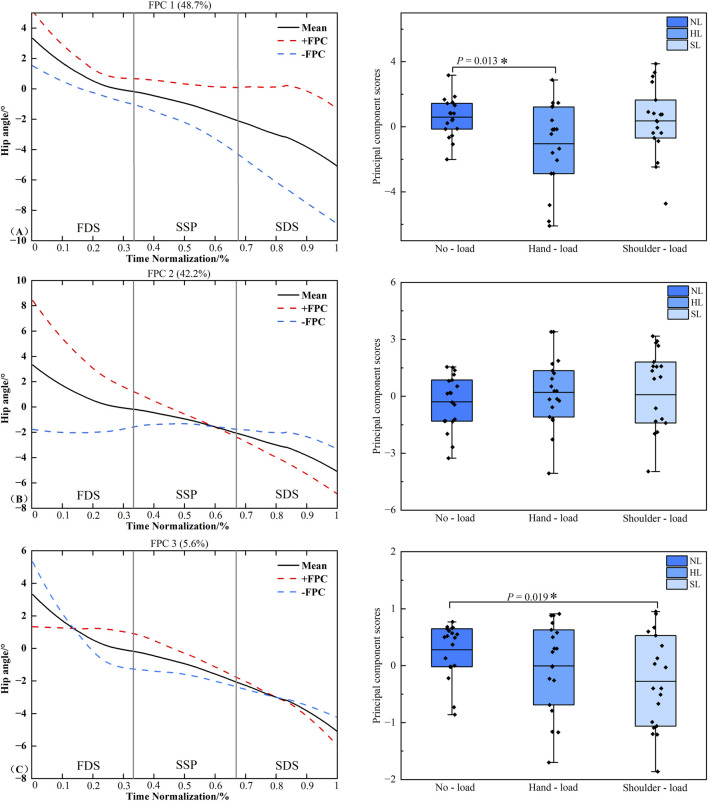
Principal component curves and scores for the hip joint frontal plane angle. Note: The solid black line represents the mean joint angle curve. The red dashed line (+) and blue dashed line (−) correspond to the mean curve plus and minus an appropriate multiple of the principal component, respectively, illustrating the mode of variability captured by that component. The corresponding box plots on the right display the scores of the principal component for each loading condition, with individual data points overlaid representing each participant’s score. **(A–C)** denote Principal Component 1 (PC1), PC2, and PC3, respectively. Asterisk (*) indicates a significant difference at P < 0.05.

Figure 8 presents the principal component analysis results of the hip joint frontal plane angle time series under different loading conditions. The kinematic time series were reduced to three principal components with eigenvalues of 4.656, 4.034, and 0.5309, explaining 48.7%, 42.2%, and 5.6% of the variance, respectively, resulting in a cumulative variance of 96.5%. The amplitude variation of PC1 was concentrated throughout the entire stance phase, while that of PC3 was primarily located during the FDS and SSP phases. Statistical analysis revealed a significant difference in PC1 scores between the unloaded and hand load conditions (*P* = 0.013, *d* = 0.831). A significant difference was also found in PC3 scores between the unloaded and shoulder-load conditions (*P* = 0.019, *d* = 0.826). No significant difference was detected in the scores of PC2 (*P* > 0.05).

### 3.7 Functional data analysis of knee joint frontal plane angle time series

**FIGURE 9:**
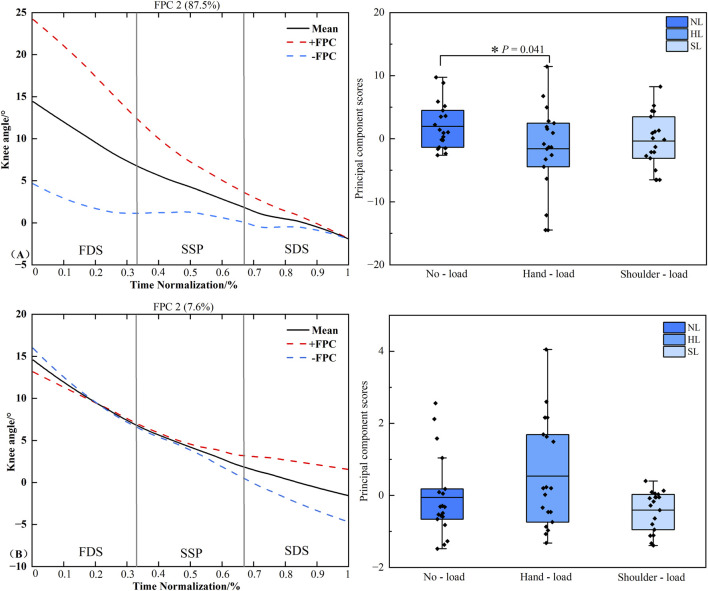
Principal component curves and scores for the knee joint frontal plane angle. Note: The solid black line represents the mean joint angle curve. The red dashed line (+) and blue dashed line (−) correspond to the mean curve plus and minus an appropriate multiple of the principal component, respectively, illustrating the mode of variability captured by that component. The corresponding box plots on the right display the scores of the principal component for each loading condition, with individual data points overlaid representing each participant’s score. **(A,B)** denote Principal Component 1 (PC1) and PC2, respectively. Asterisk (*) indicates a significant difference at P < 0.05.

Figure 9 presents the principal component analysis results of the knee joint frontal plane angle time series under different loading conditions. The kinematic time series were reduced to three principal components with eigenvalues of 26.498 and 2.293, explaining 87.5% and 7.6% of the variance, respectively, resulting in a cumulative variance of 96.5%. The amplitude variation of PC1 was primarily concentrated during the FDS, SSP phases, and the early portion of the SDS phase. Statistical analysis revealed a significant difference in PC1 scores between the unloaded and hand load conditions (*P* = 0.041, *d* = 0.653). No significant difference was detected in the scores of PC2 (*P* > 0.05).

### 3.8 Functional data analysis of ankle joint frontal plane angle time series

**FIGURE 10:**
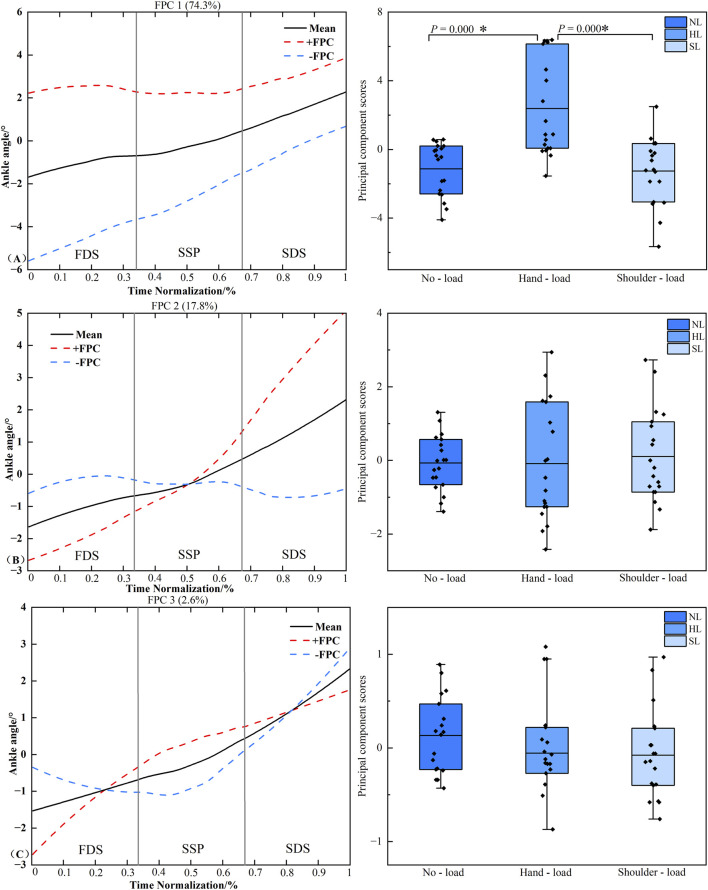
Principal component curves and scores for the ankle joint frontal plane angle. Note: The solid black line represents the mean joint angle curve. The red dashed line (+) and blue dashed line (−) correspond to the mean curve plus and minus an appropriate multiple of the principal component, respectively, illustrating the mode of variability captured by that component. The corresponding box plots on the right display the scores of the principal component for each loading condition, with individual data points overlaid representing each participant’s score. **(A–C)** denote Principal Component 1 (PC1), PC2, and PC3, respectively. Asterisk (*) indicates a significant difference at P < 0.05.

Figure 10 presents the principal component analysis results of the ankle joint frontal plane angle time series under different loading conditions. The kinematic time series were reduced to four principal components with eigenvalues of 7.282, 1.742, and 0.255, explaining 74.3%, 17.8%, and 2.6% of the variance, respectively, resulting in a cumulative variance of 94.7%. The amplitude variation of PC1 was concentrated throughout the entire stance phase of the right limb. Statistical analysis revealed significant differences in PC1 scores between the unloaded and hand load conditions (*P* < 0.001, *d* = −1.549), as well as between the hand load and shoulder-load conditions (*P* < 0.001, *d* = 1.500). No significant differences were detected in the scores of PC2 or PC3 (*P* > 0.05).

## 4 Discussion

Previous research has indicated that variability in gait parameters, such as step length and walking velocity—particularly the mean coefficient of variation (CV) of the gait cycle—is a more sensitive predictor of fall risk compared to their absolute values (Sotirakis et al., 2024). Furthermore, the trajectory of the COP during locomotion has been recognized as a key indicator of dynamic stability (Li et al., 2020). Therefore, this study employed a one-way analysis of variance to systematically evaluate the effects of different load carriage methods on gait characteristics and COP measures. The results demonstrated that the load carriage conditions significantly altered specific spatiotemporal gait parameters and reduced stability in the mediolateral direction. However, no significant effects were observed regarding anteroposterior stability, gait temporal variability, step width, or overall walking velocity and rhythm. This pattern suggests that, for healthy adults, a 15 kg load (approximately 20% of the mean participant body weight) may remain within a range that the neuromuscular system can effectively manage through compensatory mechanisms (Wei et al., 2024). To preserve macroscopic gait stability and efficiency, the nervous system appears to prioritize maintaining a consistent overall rhythm and balance control strategy. Consequently, load carriage at 20% body weight appears to induce relatively minor alterations in stair walking gait, insufficient to provoke a fundamental change in walking strategy (Zhang et al., 2018). Significant differences in single support time and second double support time were observed between both the unloaded and hand load conditions, as well as between the unloaded and shoulder-load conditions. This indicates that gait is a cyclical process of maintaining dynamic balance of the body’s center of mass. The application of an external load, regardless of its specific configuration, alters the human body’s center of mass position and inertial properties, thereby triggering corresponding postural adjustments that disrupt single-limb support capability and dynamic balance control during the propulsion phase (Gao et al., 2025). From a biomechanical perspective, the single support phase reflects the weight-bearing capacity and stability of the unilateral lower limb during the gait cycle, while the second double support phase is critical for the smooth forward transition of the body’s center of mass. Load carriage likely increases the demand on the postural control system by altering the body’s center of mass position and moment of inertia, thereby compelling the nervous system to adjust support phase timing to maintain overall stability. This finding aligns with previous studies reporting that a reduced walking speed is accompanied by prolonged duration of specific support phases (Thakurta et al., 2015; Anwer et al., 2022). Typically, a shortened single support time suggests diminished weight-bearing capacity of the lower limb. In contrast, individuals with gait abnormalities or impaired walking function often exhibit prolonged support times to enhance stability during locomotion (Wang C. et al., 2022; Liu et al., 2023). Under challenging walking conditions, the human body actively adjusts its gait pattern to maintain balance, commonly manifesting as reduced single-limb support time and extended double-support time (Zhang et al., 2018). The current study revealed that during shoulder-load carriage, participants demonstrated a significantly shorter single support time compared to other loading conditions, coupled with a markedly longer second double support time. This pattern suggests that shoulder-load carriage may pose an elevated risk of falls.

The increased step length observed during shoulder-load carriage may be attributed to the elevated body center of mass resulting from the additional load (Pitts et al., 2024). To maintain stability, the body instinctively adopts a slightly forward-inclined posture. During movement, this forward inclination causes the body’s line of gravity to more readily shift anterior to the base of support of the stance foot, generating a propulsive torque that drives the body forward. To manage this forward acceleration and maintain dynamic balance, the lower limb may need to advance by increasing step length. Consequently, shoulder-load carriage manifests as a significant increase in step length. Furthermore, the shoulder-load carriage necessitates maintaining a rigid upright torso to protect the spine, which imposes constraints on the free anterior elevation of the thigh relative to the pelvis, thereby limiting the range of hip flexion. Consequently, the body exhibits a compensatory strategy wherein, under the requirement for increased step length driven by forward inclination, it augments knee flexion to offset the restricted hip mobility. This mechanism facilitates a longer step length while ensuring spinal safety and propulsion efficiency. This hip-knee coordinated compensation finds strong support in gait-related pathological studies on individuals with femoroacetabular impingement syndrome. When the hip joint exhibits biomechanical dysfunction due to morphological abnormalities, the lower limb compensates through a coordinated pattern involving the knee and ankle joints to accomplish the walking task (Hao et al., 2025). Furthermore, this mechanism is corroborated by the functional data analysis of the hip and knee joint sagittal plane angle time series in the present study. For the PC1 of the hip joint sagittal angle, the shoulder-load condition demonstrated significantly reduced hip flexion compared to both the unloaded and hand load conditions (Figure 5A), with variability persisting throughout the right limb stance phase. Conversely, for the knee joint’s PC1, although a trend towards increased knee flexion was observed under the shoulder-load condition compared to the unloaded condition, the principal component scores did not reach statistical significance (Figure 6).

The joint angle principal component curves presented in this study were processed with continuous registration to eliminate phase variability, thereby reflecting the amplitude variation characteristics of each principal component. The solid line represents the mean joint angle curve, while the “+” curve corresponds to the mean curve plus an appropriate multiple of the principal component. The region bounded by the “+” and “-” curves illustrates the location and magnitude of the variability (Liang et al., 2017). When an individual’s principal component score is positive, their joint angle curve approximates the “+” curve, and conversely, it approximates the “-” curve when the score is negative. In the PC1 curve of the hip joint frontal plane angle, the hip abduction angle during hand load carriage was greater than that in the unloaded condition throughout the right limb stance phase, whereas the corresponding knee joint frontal plane angle was smaller compared to the unloaded condition (Figures 8, 9). This pattern suggests that when carrying a load in the right hand, the body prioritizes compensation at the proximal hip joint to maximize stability under asymmetric loading. This is achieved through increased hip abduction on the right side (via gluteal muscle contraction, causing the right leg to abduct) to control pelvic stability (Braun et al., 2025). Concurrently, the distal knee joint is stabilized by reducing unnecessary frontal plane motion, forming a more rigid support column during the stance phase. This compensatory strategy aligns with the knee’s biomechanical requirement to maintain multi-plane dynamic stability during weight-bearing (An et al., 2023). PCA of the ankle joint frontal plane angle time series revealed that the variability in the ankle’s frontal plane movement pattern was primarily captured by the PC1. This variability was distributed throughout the entire right limb stance phase. During the stance phase, the ankle joint frontal plane angle under the hand load condition was significantly greater than that under both the unloaded and shoulder-load conditions (Figure 10). This likely occurs because during hand load carriage, the body’s center of gravity shifts toward the load-bearing side. To maintain frontal plane stability during the single-leg support phase, the right ankle joint must generate a greater inversion moment to counteract the tendency of the body to tilt toward the contralateral side. This compensatory adjustment is directly manifested as an increased inversion angle of the ankle joint in the frontal plane, which aligns with previous findings regarding adaptive adjustments in distal joints induced by asymmetric loading (Hao et al., 2025). Furthermore, the COP findings are corroborated by the gait analysis. COP displacement is widely used to assess postural stability, with poorer stability typically manifesting as increased COP displacement distance. The significant differences in COP displacement in the mediolateral (frontal plane) direction across different load carriage conditions indicate that the interference of load carriage on postural control is direction-specific, primarily affecting the balance system responsible for resisting lateral instability (Wang Y. et al., 2022; Lehmann et al., 2017). The significant difference between the unloaded and hand load conditions reflects the pronounced disruption of the balance system by unilateral loading. In contrast, the difference between the hand load and shoulder-load conditions underscores that shoulder-load carriage confers an advantage over hand load in maintaining mediolateral stability. Together, these biomechanical insights may inform the design of task-specific load carriage recommendations and contribute to a refined understanding of stability mechanisms, which are foundational for developing targeted fall prevention strategies.

## 5 Conclusion

This study employed FDA to elucidate the specific neuromuscular compensation mechanisms elicited by different load carriage methods during stair walking. A key finding is that the biomechanical risk profile is critically dependent on load configuration. The shoulder load induced a fundamental gait phase alteration—shortened single support and prolonged double support—indicating a systematic stability challenge necessitating global strategy adjustment. In contrast, the hand load triggered localized frontal-plane adaptations. These findings underscore that unilateral load carriage, especially on the shoulder, presents pronounced stability risks for healthy young males. Therefore, this study provides a biomechanical basis for formulating tailored ergonomic guidelines. It should be noted that fall risk was inferred from stability measures, not directly assessed via balance-loss events, and the findings are limited to dominant leg.

## 6 Limitations

While this study employed FDA to systematically investigate the effects of different load carriage methods on gait variability during stair walking, several limitations must be acknowledged. First, the sample size was small and limited to healthy young males, restricting statistical power and generalizability. Second, fPCA was applied separately to each joint and plane; a holistic analysis integrating all joints might reveal different coordination patterns. Third, only a single load magnitude was tested, leaving dose–response relationships unexplored. Fourth, no formal sensitivity analysis was conducted to verify the robustness of the principal components against outliers. Finally, while fPCA identified key variability modes, their physiological interpretation remains inferential. Future studies should address these points with larger, more diverse cohorts, multi-load designs, robustness checks, and complementary methods such as electromyography.

## Data Availability

The original contributions presented in the study are included in the article/supplementary material, further inquiries can be directed to the corresponding authors.

## References

[B1] An Y. Zhang J. Li H. Yuxiao Z. Guoliang X. Kun G. (2023). Synergistic characteristics of lower extremity muscles with unilateral knee flexion limitation. Chin. J. Tissue Eng. Res. 27 (29), 4704–4711. 10.12307/2023.658

[B2] Anwer S. Li H. Antwi-Afari M. F. Umer W. Mehmood I. Wong A. Y. L. (2022). Effects of load carrying techniques on gait parameters, dynamic balance, and physiological parameters during a manual material handling task. Eng. Constr. Archit. Manag. 29 (9), 3415–3438. 10.1108/ecam-03-2021-0245

[B3] Boffey D. Harat I. Gepner Y. Frosti C. L. Funk S. Hoffman J. R. (2019). The physiology and biomechanics of load carriage performance. Mil. Medicine 184 (1-2), e83–e90. 10.1093/milmed/usy218 30252089

[B4] Braun L. Mai P. Hipper M. Denis Y. Helwig J. Anedda B. (2025). Managing lower extremity loading in distance running by altering sagittal plane trunk leaning. J. Sport Health Sci. 14, 100985. 10.1016/j.jshs.2024.100985 39251186 PMC11809138

[B5] Conway Z. J. Silburn P. A. Blackmore T. Cole M. H. (2017). Evidence of compensatory joint kinetics during stair ascent and descent in Parkinson’s disease. Gait and Posture 52, 33–39. 10.1016/j.gaitpost.2016.11.017 27863279

[B6] Donoghue O. A. Harrison A. J. Coffey N. Hayes K. (2008). Functional data analysis of running kinematics in chronic achilles tendon injury. Med. Science Sports Exercise 40 (7), 1323–1335. 10.1249/mss.0b013e31816c4807 18580414

[B7] Gao T. Tang B. Wu X. (2025). Research review and outlook on human posture control. Front. Nat. Sci. 1 (4). 10.63887/fns.2025.1.4.4

[B8] Halsey L. G. Watkins D. A. R. Duggan B. M. (2012). The energy expenditure of stair climbing one step and two steps at a time: estimations from measures of heart rate. PLoS One 7 (12), e51213. 10.1371/journal.pone.0051213 23251455 PMC3520986

[B9] Hao Y. Ren S. Zhu Y. Miao X. Xu Y. (2025). Three-dimensional lower limb kinematics and kinetics in femoroacetabular impingement syndrome (FAIS) patients with and without borderline developmental dysplasia of the hip (BDDH) during level walking. BMC Musculoskelet. Disord. 26 (1), 488. 10.1186/s12891-025-08727-4 40380344 PMC12082895

[B10] Jolliffe I. (2002). Principal component analysis. Berlin: Springer Science&Business Media.

[B11] Kováčiková Z. Sarvestan J. Zemková E. (2021). Age-related differences in stair descent balance control: are women more prone to falls than men? PloS One 16 (1), e0244990. 10.1371/journal.pone.0244990 33411803 PMC7790224

[B12] Lehmann T. Paschen L. Baumeister J. (2017). Single-leg assessment of postural stability after anterior cruciate ligament injury: a systematic review and meta-analysis. Sports Medicine-Open 3 (1), 32. 10.1186/s40798-017-0100-5 28853022 PMC5574832

[B13] Leroy A. Marc A. Dupas O. Rey J. L. Gey S. (2018). Functional data analysis in sport science: example of swimmers’ progression curves clustering. Appl. Sci. 8 (10), 1766. 10.3390/app8101766

[B14] Li B. Xiang Q. Zhang X. (2020). The center of pressure progression characterizes the dynamic function of high-arched feet during walking. J. Leather Sci. Eng. 2 (1), 1. 10.1186/s42825-019-0016-6

[B15] Liang Y. Shen Q. Liu L. (2017). Application of the functional principal component analysis (FPCA) model: a case study of population regulation in Beijing's industries. World Surv. Res. (02), 29–33. 10.13778/j.cnki.11-3705/c.2017.02.006

[B16] Lin H. Yan B. Liu Z. Xu C. Liang H. (2012). Quantitative methods and research status of motor coordination in sport skills. China Sport Sci. 32 (03), 81–91. 10.16469/j.css.2012.03.011

[B17] Liu S. Yu H. Wang Z. Dai P. (2023). Correlation analysis of balance function with plantar pressure distribution and gait parameters in patients with cerebral infarction in the basal ganglia region. Front. Neurosci. 17, 1099843. 10.3389/fnins.2023.1099843 36908774 PMC9998687

[B18] Ozgul B. Akalan N. E. Kuchimov S. Uygur F. Temelli Y. Polat M. G. (2012). Effects of unilateral backpack carriage on biomechanics of gait in adolescents: a kinematic analysis. Acta Orthop. traumatologica turcica 46 (4), 269–274. 10.3944/aott.2012.2678 22951758

[B19] Pitts J. Komisar V. Elmblad K. Smith A. Verbrigghe D. Siko C. (2024). Influences of backpack loading on recovery from anterior and posterior losses of balance: an exploratory investigation. Appl. Ergon. 117, 104236. 10.1016/j.apergo.2024.104236 38237306

[B20] Ramsay J. (2018). Functional data analysis. Available online at: http://www.psych.mcgill.ca/misc/fda/software.html (Accessed 07 February 2018).

[B21] Ramsay J. O. Li X. (1998). Curve registration. J. R. Stat. Soc. Ser. B Stat. Methodol. 60 (2), 351–363. 10.1093/oxfordhb/9780199568444.013.9

[B22] Richter C. Marshall B. Moran K. (2014). Comparison of discrete-point vs. dimensionality-reduction techniques for describing performance-related aspects of maximal vertical jumping. J. Biomechanics 47 (12), 3012–3017. 10.1016/j.jbiomech.2014.07.001 25059895

[B23] Ryan W. Harrison A. Hayes K. (2006). Functional data analysis of knee joint kinematics in the vertical jump. Sports Biomech. 5 (1), 121–138. 10.1080/14763141.2006.9628228 16521626

[B24] Simpson K. M. Munro B. J. Steele J. R. (2012). Effects of prolonged load carriage on ground reaction forces, lower limb kinematics and spatio-temporal parameters in female recreational hikers. Ergonomics 55 (3), 316–326. 10.1080/00140139.2011.642004 22409169

[B25] Sotirakis C. Brzezicki M. A. Patel S. Conway N. FitzGerald J. J. Antoniades C. A. (2024). Predicting future fallers in Parkinson’s disease using kinematic data over a period of 5 years. NPJ Digital Medicine 7 (1), 345. 10.1038/s41746-024-01311-5 39638907 PMC11621420

[B26] Su B. Jiang J. Tang Q. Sheng M. (2017). Human dynamic action recognition based on functional data analysis. Acta Autom. Sin. 43 (05), 866–876. 10.16383/j.aas.2017.c160120

[B27] Thakurta A. G. Iqbal R. Bhasin H. V. (2015). “Comparative analysis of gait kinematics of male and female construction workers while carrying asymmetric loads in different modes,” in 2015 International Conference on Industrial Engineering and Operations Management (IEOM) (IEEE), 1–5. 10.1109/ieom.2015.7093754

[B28] Wang H. Frame J. Ozimek E. Leib D. Dugan E. L. (2013). The effects of load carriage and muscle fatigue on lower-extremity joint mechanics. Res. Quarterly Exercise Sport 84 (3), 305–312. 10.1080/02701367.2013.814097 24261009

[B29] Wang C. Zhang M. Zhou J. Lao K. (2022). Early gait changes after total hip arthroplasty through direct anterior approach and posterolateral approach. Chin. J. Tissue Eng. Res. 26 (03), 359–364. 10.12307/2022.059

[B30] Wang Y. Zhu T. Lin J. Zhang N. Wei H. Chen X. (2022). Journal of medical biomechanics. J. Med. Biomechanics 37 (03), 531–537. 10.16156/j.1004-7220.2022.03.023

[B31] Wang H. H. Tsai W. C. Chang C. Y. Hung M. H. Tu J. H. Wu T. (2023). Effect of load carriage lifestyle on kinematics and kinetics of gait. Appl. Bionics Biomechanics 2023 (1), 8022635. 10.1155/2023/8022635 36816755 PMC9931482

[B32] Warmenhoven J. (2024). Over 30 years of using functional data analysis in human movement. What do we know, and is there more for sports biomechanics to learn? Sports Biomech., 1–32. 10.1080/14763141.2024.2398508 39475398

[B33] Warmenhoven J. Harrison A. Robinson M. A. Vanrenterghem J. Bargary N. Smith R. (2018). A force profile analysis comparison between functional data analysis, statistical parametric mapping and statistical non-parametric mapping in on-water single sculling. J. Sci. Med. Sport 21 (10), 1100–1105. 10.1016/j.jsams.2018.03.009 29650339

[B34] Wei J. Huo H. Bai X. Zheng D. (2024). Functional differentiation of bilateral feet in young women walking with increasing weight bearing. Chin. J. Sports Med. 43 (04), 258–265. 10.16038/j.1000-6710.2024.04.007

[B35] Zhang T. Zhang J. Ji R. Lv Z. Li Z. (2018). A comparative analysis of the influence of different loading ways on the walking stability of the elderly. Chin. J. Sports Med. 37 (12), 1005–1010. 10.16038/j.1000-6710.2018.12.006

[B36] Zhang B. Zhou X. Xi J. (2019). Functional data analysis of characteristics in the development of galloping performance. China Sport Sci. 39 (11), 48–56. 10.16469/j.css.201911005

